# Efficacy of acupuncture and electroacupuncture in patients with nonspecific low back pain: study protocol for a randomized controlled trial

**DOI:** 10.1186/s13063-015-0850-7

**Published:** 2015-10-15

**Authors:** Josielli Comachio, Mauricio Oliveira Magalhães, Thomaz Nogueira Burke, Luiz Armando Vidal Ramos, Gabriel Peixoto Leão Almeida, Ana Paula M. C. C. Silva, Sarah Rúbia Ferreira de Meneses, Jecilene Rosana Costa-Frutuoso, Cinthia Santos Miotto Amorim, Amélia Pasqual Marques

**Affiliations:** Department of Physical Therapy, Communication Science & Disorders, Occupational Therapy, University of São Paulo, São Paulo, Brazil; Federal University of Amapá - Campus Binacional do Oiapoque, Amapá, Brazil; Department of Physical Therapy, Medical School, Federal University of Ceará, Fortaleza, Brazil

**Keywords:** Electroacupunture, acupuncture, low back pain, physiotherapy

## Abstract

**Background:**

Previous studies have shown that acupuncture and electroacupuncture (EA) are effective in the treatment of patients with low back pain. However, there is little evidence to support the use of one intervention over the other. The aim of this study is to compare the effect of acupuncture and electroacupuncture in the treatment of pain and disability in patients with chronic nonspecific low back pain.

**Methods/design:**

The study design is a randomized controlled trial. Patients with nonspecific chronic low back pain of more than three months duration are recruited at Rehabilitation Center of Taboao da Serra - SP (Brazil). After examination, sixty-six patients will be randomized into one of two groups: acupuncture group (AG) (n = 33) and electroacupuncture group (EG) (n = 33). Interventions will last one hour, and will happen twice a week for 6 weeks. The primary clinical outcomes will be pain intensity as measured and functional disability. Secondary outcomes: quality of pain, quality of life. perception of the overall effect, depressive state, flexibility and kinesiophobia. All the outcomes will be assessed will be assessed at baseline, at treatment end, and three months after treatment end. Significance level will be determined at the 5 % level. Results of this trial will help clarify the value of acupuncture and electroacupuncture as a treatment for chronic low back pain and if they are different.

**Discussion:**

Results of this trial will help clarify the value of acupuncture needling and electroacupuncture stimulation of specific points on the body as a treatment for chronic low back pain.

**Trial Registration:**

Clinicaltrials.gov: NCT02039037. Register October 30, 2013.

## Background

Low back pain is a serious public health and socioeconomic problem worldwide; it relates to levels of absenteeism at work [1, 7] and affects quality of life and functional performance [1]. It also entails enormous social and economic costs. There are wide varieties of treatment options for chronic low back that are endorsed by clinical practice guidelines [3, 7, 10]. In an attempt to reduce the impact associated with chronic nonspecific low back pain, certain treatments have been recommended by The European Guidelines for the Management of Chronic Low Back Pain as effective in the treatment of the condition, such as manipulation/mobilization, acupuncture, yoga, massage therapy back school, and multidisciplinary treatment [1]. General advice on the self-management of nonspecific LBP should include recommendations to remain active and encouragement to return to normal activities as soon as possible because many individuals evolve to significant levels of functional disability due to fear of movement [9].

Acupuncture is based on the concepts of traditional Chinese medicine (TCM) and is one of the oldest forms of complementary therapy. During the past quarter of a century numerous systematic reviews have investigated the effectiveness of acupuncture in the management of LBP, but review conclusions are sometimes contradictory and often limited by the number and quality of the included studies [3]. To understand the physiologic mechanism of acupuncture, some studies report that the technique causes inhibition in the dorsal horn, which can activate or inhibit certain points of the body that stimulate the release of opioids such as serotonin. Further investigation explored the role of central neurotransmitters in mediating acupuncture analgesia, including capicolaminas and cerotoninas [29, 31]. When released these neurotransmitters produce various effects, such as analgesic, muscle relaxant, anti-inflammatory, mild anxiolytic and antidepressant effects.

Electroacupuncture (EA) is the application of electrical stimulation to acupuncture needles and has been widely practiced and has been indicated in some cases where treatment with traditional acupuncture (or another technique) has failed. This technique is used because it can improve the electrical stimulus of certain physiological reactions and/or other produce different may obtain a faster analgesic and anesthetic that manual acupuncture, low frequencies are indicated for the use of electroacupuncture in patients with LBP [13, 15]. One of the main advantages of using EA in clinical practice or acupuncture research is its ability to set stimulation frequency and intensity. Lewith [16] compared the efficacy of transcutaneous electrical nerve stimulation (TENS) with EA in the treatment of chronic lower back pain. The study showed that EA produces a greater reduction in pain scores than TENS. Thomas and Lundberg [22, 24] also demonstrated that the effectiveness of low frequency EA was effective in reducing chronic LBP.

A recent systematic review concluded that acupuncture and EA has become a popular and complementary practice in the treatment of LBP [14]. The availability and practicability of acupuncture are also important factors to consider; the advantages of acupuncture are that it is simple, convenient and has few contraindications. However, it is still not possible to verify its effectiveness compared to EA in patients with LBP, but rather as an adjunct to other forms of therapy. Furthermore, most studies evaluate only aspects of short-term pain. Thus, it is suggested that more studies be conducted with the aim of verifying the effectiveness of acupuncture and EA in the medium term, in addition to investigating the effects of treatments on psychosocial aspects.

### Study aim

The aim of this study is to report the study protocol used to investigate the effect of EA and acupuncture treatment in reducing the symptoms of nonspecific LBP.

## Methods and design

This study will be a randomized controlled trial, approved by the Ethics Committee of the School of Medicine of the University of Sao Paulo (protocol study 350/13), and funded by the Coordenação de Aperfeiçoamento de Pessoal de Nível Superior (CAPES). To examine the longevity of any intervention effects, measurements will be taken before treatment, after 6 weeks of treatment and 3 months after the intervention. This randomized controlled clinical trial began recruitment on January 16, 2014 and the anticipated completion date is April 2015. We will recruit 66 patients at the Rehabilitation Center of Taboao da Serra, Sao Paulo, Brazil.

After submitting informed consent and being randomized, patients will receive 12 sessions of Chinese acupuncture (acupuncture group (AG)) and EA treatment (EA group (EG)) lasting one hour, twice a week for 6 weeks. Patients will be asked to accept assessments at baseline and at the end of the first and second weeks of the treatment phase. The protocol for the AG is described in Table 1.

**Table 1:** Protocol for acupoints used in the project

Acupoints	Location	Major indication e Function
BV 41(Zulinqi)	On the dorsum of the foot, the proximal angle between the fourth and fifth metatarsal bone on the lateral depression of the extensor tendon of the little finger	Relieves joint stiffness and muscle spasms
TA 5 (Waiguan)	Near the dorsal wrist crease between the radius bone and ulna	Relaxes and strengthens tendons
E 36 (Zusanli)	Three inches below the patella between the anterior tibia and the extensor digit rum longs muscle	Tiredness, fatigue caused by weakness and irritability
H 3 (Shaohai)	With the elbow flexed, between the inner end of the cubital crease and the epicondyle of the humerus	Soothes and strengthens the mind
LI 4 (Hegu)	The dorsal side of the hand between the first and second metacarpal bone of the middle dorsal interosseous muscle, opening the thumb and forefinger in the middle of the junction line between the first and the second metacarpal bone	Spasm in fingers
R7 (Fuliu)	Two inches above the point R3 on the anterior medial edge of the soleus muscle	Leg muscle atrophy; swelling
GV 4 (Mingmen)	The dorsal midline in the depression below the spinous process of vertebra L2	Strengthens the lower back and knees
BP 6 (Sanyinjiao)	Three inches above the medial malleolus in the posteromedial border of the tibia	Pain, weakness and imbalance; motor and mental asthenia
B23 Shenshu	B23: one and a half inches toward the lower border of the spinous process of vertebra L2, 2 cm lateral to the midline.	Bone and kidney problems
B30 Baihuanshu	B30: one and a half inches toward the midline of the spine on the foramen of the fourth posterior sacral level	Hip pain; feeling cold in the lower back
B58 Feiyang	B58: seven inches above the heel on the lateral side of the tendon of the gastrocnemius muscle	Weakness of the leg muscles; leg pain, back pain
B60 Kunlun	B60: between the Achilles tendon and the edge of the lateral malleolus of the ankle on the highest point of the malleolus level	Headache strengthens the lumbar and thoracic region

Acupuncture points chosen will be selected based on the characteristics of patients and literature suggested (Wang KM; George SZ)

### Patients

#### Inclusion criteria

Patients are eligible for inclusion if they were 20–60 years old, with nonspecific chronic LBP for more than 3 months duration and a minimum pain intensity score of 3 in the 11-point pain numerical rating scale.

#### Exclusion criteria

Patients will be excluded if they had previously had surgery to the spinal column, known or suspected serious spinal pathology (e.g., fractures, tumors, inflammatory or rheumatologic disorders, or infective diseases of the spine), severe cardiopulmonary disease, rheumatic disease, were pregnant, had a pacemaker or metal implants, or did not understand the written and spoken Portuguese language [26]. All participants will be invited to sign the participant consent form. The methodoly of this study is based on standards established under the Consolidated Standards of Reporting Trials (CONSORT) [18] and Revised Standards for Reporting Interventions in Clinical Trials of Acupuncture (SCRICTA) [20].

### Procedures

We will recruit patients with chronic LBP (with symptoms of at least 3 months duration) who are seeking care for their problem. The reviews will take place in three stages: before treatment sessions begin, at the end of treatment (6 weeks) and 3 months after the end of the sessions. The envelopes will be opened sequentially by the treating physiotherapists, who will immediately provide the first session of treatment to the patients. Acupuncture points chosen will be selected based on the characteristics of patients and the relevant literature [11, 27].

The treatment protocol for the opening of the points will include the Daí Mai (VB41-TR5) meridian, points of local action and symptomatic points chosen by probing. The EG will be treated with electrical stimulation toning of 10 Hz for 10 minutes at the symptomatic points Zhenjin B23, Baihuanshu B30 and bilateral Mingmen VG4 (Table 1).

### Randomization procedures

Before the treatment begins, the patients will be randomly allocated to their respective intervention groups. The random allocation sequence will be implemented by one of the researchers not involved with recruiting and assessing the patients, and will be generated on Microsoft Excel 2013 software. This random allocation sequence will be inserted into sequentially numbered, opaque, sealed envelopes (to ensure that allocation is concealed from the assessor). The envelopes will be opened by the physical therapist who will treat the patients.

### Outcome measures

The primary outcomes will be pain intensity and disability. The secondary outcomes will be quality of pain, quality of life, perception of overall effect, depression, flexibility and kinesiophobia. All scales and questionnaires have been translated into Brazilian Portuguese and their properties clinimetrically tested [6, 8, 19].

#### Primary outcomes

##### Pain intensity

Pain intensity will be assessed using the numerical rating scale (NRS). The NRS is an 11-point scale ranging from 0 to 10 in which 0 indicates an absence of pain and 10 indicates unbearable pain. Participants will be asked to rate their average pain levels for the week prior to assessment [8].

##### Disability

The Roland Morris disability questionnaire will be used to assess functional disability due to LBP. This questionnaire consists of 24 questions that focus on the regular activities of daily life. Each affirmative answer corresponds to 1 point, and the final score is determined by the total number of points − the total score ranges from 0 to 24, and higher scores reflect increased disability. Scores above 14 indicate severe impairment [6, 19].

#### Secondary outcomes

##### Quality of pain

The McGill pain questionnaire provides a multidimensional assessment of pain. It consists of 78 descriptors of the quantity and quality of pain which are grouped into four major domains (sensory, affective, evaluative and miscellanea) and 20 subdomains to which intensity values are assigned scores on a scale of 1 to 5. The questionnaire is used to describe pain experience, and the score corresponds to the sum of the aggregated values. Maximum scores will be as follows: sensorial = 41, affective = 14, evaluative = 5, miscellanea = 17, and total = 77. The index of pain assessment is the sum of added values, and each word chosen in each dimension is the maximum score for each category [25].

##### Depression

The Beck depression inventory (BDI) is an instrument that assesses the severity of depression. The original rating scale consists of 21 items that assess symptoms and attitudes that vary on a scale of 0 to 3. The items in the inventory evaluate the following attitudes and symptoms: sadness, pessimism, sense of failure, lack of satisfaction, guilt, sense of punishment, self-depreciation, self-reproach, suicidal thoughts, crying, irritability, social withdrawal, indecisiveness, distortion of body image, inability to work, sleep disturbance, fatigue, loss of appetite, weight loss, somatic preoccupation and decreased libido. The scores for depression are: normal (<15), mild (16–20) and severe (>20). Higher values indicate a greater severity of depressive symptoms [2, 28].

##### Quality of life

The short form health survey questionnaire (SF-36) assesses health-related qualify of life. It consists of 36 questions grouped in 8 domains: vitality (4 items), physical functioning (10 items), bodily pain (2 items), general health (5 items), physical role (2 items), emotional role (3 items), social functioning (2 items) and mental health (5 items). For each domain, scores range from 0 to 100 and higher scores reflect a better quality of life. Only the physical and emotional domains will be used in this study [4].

##### Global perceived effect

The global perceived effect scale is an 11-point scale ranging from –5 to 5, where –5 indicates vastly worse, 0 indicates no change and 5 indicates completely recovered. For all measures of global perceived effect (at baseline and in all follow up), participants will be asked: “Compared to when this episode first started, how would you describe your lower back pain these days?” A higher positive score indicates greater recovery and 9 of 12 negative scores indicate a worsening of symptoms. This outcome will be measured at baseline, after 6 weeks of treatment and 3 months after the intervention [5].

##### Kinesiophobia

The Tampa scale of kinesophobia (TSK), which was developed to measure the fear of movement due to chronic LBP, is a self-applied questionnaire consisting of 17 items. Each question has four response options (strongly disagree, disagree, agree and strongly agree) with scores ranging from 1 to 4 points, respectively. The scores of items 4, 8, 12 and 16 are inverted and the total score is the sum of the items, which ranges from 17 to 68 points. Increased scores reflect an increased fear of movement [23].

##### Flexibility

The flexibility of the posterior chain will be assessed by the 3 ° finger test. Participants will be asked to keep their knees fully extended and to flex their torsos toward the floor with their arms relaxed around the head. Individuals who can reach to less than 10 cm from the ground and who can touch the floor will be classified as having normal flexibility, and those who cannot reach beyond 10 cm from the floor will be classified as having reduced flexibility [17].

### Intervention

**Fig. 1:**
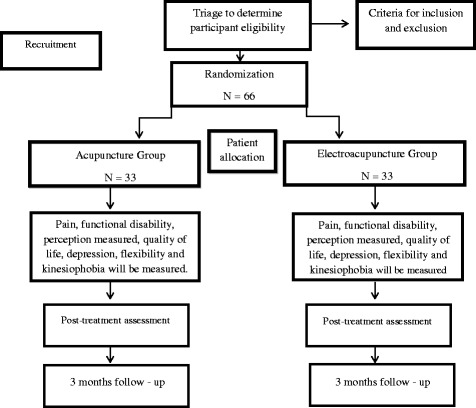
Flow diagram of study design showing the randomization of participants. Patients will receive 12 sessions of Chinese acupuncture or electroacupuncture treatment lasting one hour, twice a week for 6 weeks

1. The AG will be treated with 0.20 × 15 Manufacture: Dong Bang, Source: Korea. Acupuncture needles at specific points (Table 1) for 40 minutes.
2. The EG will be treated with 30 minutes of acupuncture, and EA stimulation will be used at 10 Hz and amplitude of up to 10 mA (individually adjusted according to the tolerance of the subject) in symptomatic points (Fig. 1) with the Accurate Pulse 585 applied for 10 minutes with direct current (intermittent).

The treatments in both groups will be administered over 6 weeks (twice a week for 12 treatment sessions) by a physiotherapist acupuncturist with experience. All patients will be directed not to seek any other type of care or treatment for their LBP for the duration of this study; they will be allowed to maintain their regular activities, which will be also monitored throughout the treatment sessions. Patients will be instructed to stay in the lateral decubitus position and to wear comfortable clothing for treatment.

### Data analysis

The sample size calculation will be performed to detect a difference of 2 points in pain intensity as measured by the NRS (estimated standard deviation = 1.9 points) and four points in functional disability as measured by the Roland Morris questionnaire (estimated standard deviation = 4.9 points). Specifications will be based on α = 0.05, statistical power of 80 % and follow up loss of 15%. Following these parameters, 33 patients will be placed in each group.

#### Sample size calculation

Sample size was defined in order to detect a 2-point difference between groups on the pain intensity outcome measured by the Pain Numerical Rating Scale, assuming a standard deviation of 1.9 points [23]. We also sought power to detect a 4-point difference in functional disability measured by the Roland Morris Disability Questionnaire, with an estimated standard deviation of 4.9 points [24, 25]. Power was defined as 80% for an alpha of 5% and attrition (drop-outs) of 15%. Accordingly, 33 participants per group will be needed.

#### Statistical analysis

Data normality will be tested through visual inspection of histograms. The statistical analysis of our study will follow intention-to-treat principles. Repeated measures analysis of variance (ANOVA) will be used to investigate the effect of treatment (Acupuncture vs Electroacupuncture), time (baseline, post-treatment, 3 months follow up), and interaction terms between treatment group versus time. If differences between groups were identified, the Turkey-test for multiple comparison will be conducted. Two-sided paired t tests will use for within-group comparisons (comparing baseline to follow up). If we found non-normally distributed data, we will use the Kurtosis-Wallis test. For all of these analyses, we will use the SPSS version 21 software (SPSS Inc, Chicago, Illinois).The confidence interval will be established at 95%, and the significance level at 0.05.

The relative gain (RG) with treatment will be calculated with the following equation:

$$ R{G}_i=\frac{\left( Baselin{e}_i-En{d}_i\right)\times 100}{Baselin{e}_i} $$

#### Ethics and data security

This trial was approved by the Ethics Committee of the School of Medicine of the University of Sao Paulo (protocol study 350/13). All patients will be asked to provide written, informed consent prior to randomization, using standard forms. Data access and storage will be in accordance with the National Health and Medical Research Council guidelines. This trial is registered on ClinicalTrials.gov (a service of the US National Institutes of Health) under the number NCT02039037 (October 30^th^, 2013).

## Discussion

The results of this study will contribute to a better understanding of the effectiveness of Electroacupunture and acupuncture in patients with chronic LBP in the short and medium term. The results of this study may help physical therapists and acupuncturists in their clinical decision making and to detect relevant clinical treatment with a low risk of bias. To increase the clinical relevance of the test results.

This trial was designed to reproduce the intervention exactly as it is currently recommended by TCM as well as guidelines [1, 10, 30] on the treatment of patients with chronic LBP, and we hope that this study will help to reduce pain and disability. Studies have already shown the effectiveness of acupuncture as an intervention in patients with LBP compared with placebo-controlled groups [2, 4, 9, 28]: in our study both techniques are applied separately, thus we can evaluate the effects separately; this has not been previously reported, and there are currently no studies demonstrating whether one technique is more effective than the other. There is currently no answer to the question: “Is electroacupuncture better than manual acupuncture?” There is a tendency, among modern acupuncturists, to consider this a truism, but there is no scientific evidence to back up this statement. More research is necessary, specifically designed to respond that question.

The study will contribute to clinical practice by providing evidence that will help guide decisions about the appropriate treatment of patients with chronic LBP and also may contribute a solution to the difficult problem of the chronicity of acute pain, and promote the clinical application of EA our acupuncture analgesia. The results will be published once the study is completed.

### Study limitations

The main limitation of the present study is the inability to blind the participants and the therapist with regard to treatment allocation. Also a possible limitation is the fact that we do not include a treatment control group that would allow for the evaluation of the absolute effects of both interventions.

## Trial status

We are currently recruiting participants.

## Abbreviations

AG: acupuncture group
BDI: Beck depression inventory
CAPES: Coordenação de Aperfeiçoamento de Pessoal de Nível Superior
CONSORT: Consolidated Standards of Reporting Trials
EA: electroacupuncture
EG: electroacupuncture group
LBP: low back pain
NRS: pain numeric scale
SF-36: short form health survey questionnaire
SPSS: Software statistical package for the social sciences
STRICTA: Revised Standards for Reporting Interventions in Clinical Trials of Acupuncture
TCM: traditional Chinese medicine
TENS: transcutaneous electrical nerve stimulation
TSK: Tampa scale of kinesiophobia
